# Identification and Illustration of Means to a Critical Assessment of Music and Health Research Literature

**DOI:** 10.3390/healthcare11060807

**Published:** 2023-03-09

**Authors:** Jahnusha Shriraam, Gilles Comeau, Mikael Swirp

**Affiliations:** Music and Health Research Institute, University of Ottawa, Ottawa, ON K1N 6N5, Canada

**Keywords:** music therapy, music in medicine, critical appraisal, music and health

## Abstract

In 2019, the WHO released a scoping review investigating art therapies in evidence-based healthcare practices to identify and understand the gaps in the literature. However, the studies curated were not evaluated for their quality. To address this limitation, several assessment tools to critically appraise music-based research studies that investigate therapies pertaining to preventative healthcare were investigated. Two critical appraisal tools were selected for their robustness and appropriateness for the studies in question: the Joanna Briggs Institute checklists and the Music-Based Intervention Guidelines. These tools were tested by two assessors on a total of 23 music studies from the Preventative and Prevention Health section of the WHO report. Based on the requirements for each critical appraisal tool, seven studies received a full assessment utilizing both checklists. Of these seven, two studies scored on the higher index, indicating that the studies followed a detailed methodology to provide concrete and accurate results. The findings of the study highlighted the limitations of study designs and music-based interventions. With this information, beneficial recommendations for future research in this domain are provided to improve the quality of research, ensuring its place in evidence-based healthcare practices.

## 1. Introduction 

Music has become a popular prescription for improving an individual’s health. Part of the appeal stems from the fact that music interventions are non-invasive and are considered low risk, making it a desirable and unique treatment option for patients and their families. The classic notion surrounding the concept of music therapy is the idea of using music as a therapeutic means of treating medical problems [1]. However, new and innovative research is starting to delve into a new concept known as “music as medicine”, investigating the relationship of sonic properties of music at the cellular level [1]. Despite the growing literature, there remains a prominent gap in the translation process of research evidence to the development of health practices and policies. To bridge this gap, a scoping review was published by Fancourt and Finn through the World Health Organization (WHO) in 2019 [2]. 

The scoping review by WHO explored the role of the arts in improving health and wellbeing by gathering studies to provide evidence for policy considerations [2]. Studies were included if a form of “art” was utilized as an intervention. The scoping review’s definition of arts included music, visual art, drama, dance, literature, cultural arts and online/digital arts [2]. A total of 962 studies were included within the WHO scoping review, with over 200 studies as systematic reviews, meta-analyses or meta-synthesis [2]. However, given the vast number of studies collected, Fancourt and Finn failed to analyze the studies for their quality, and the reliability and validity of the interventions administered. The significance behind analyzing a study for these components relates to the importance of high-quality research in evidence-based practice in the healthcare setting. 

In response to this limitation, a second report was released by the Department of Digital, Culture, Media and Sport (DCMS) in April 2020 [3]. This evidence summary for policy was created by Fancourt and colleagues to assess selected topics within the WHO scoping review [2], against a quality assessment framework known as FORM [3]. FORM, created by the National Health and Medical Research Council (NHMRC) of Australia, is a structured process used to analyze large bodies of research for internal and external validity against five criteria: quality and quantity of evidence base, consistency of findings, potential clinical impact, generalizability, and applicability [3,4]. However, for the purpose of their report, Fancourt and colleagues modified the FORM framework by altering the evidence matrix to account for non-clinical policy guideline development [3]. Of the many topics explored within the WHO scoping review [2], special attention was given to social outcomes, youth development, and prevention of mental and physical illnesses in adulthood [3]. The report was divided into three sections, with each section outlining the number of randomized control trials, and quantitative and qualitative studies. At the end of each section, a summary of the findings was provided in addition to a set of grading recommendations based on the five criteria mentioned above. The grading was denoted by letter grades, with grades A and D being the highest and lowest, respectively [4]. Grades A and B denoted that the research evidence could be trusted to guide clinical practice, while Grades C and D were used to identify research evidence that should be interpreted with caution and applied carefully in individual/organizational circumstances [4].

While the report addressed a part of the issue regarding quality of evidence, a series of concerns were addressed by Clift and colleagues in 2021, through their critique of the research conducted by both WHO and DCMS [5]. The critical commentaries made by Clift and colleagues suggested that the WHO and DCMS reports failed to account for several factors. Firstly, the research presented did not include the current literature available, essentially omitting bodies of evidence that should have been included in the review [5]. Based on the analysis of the existing research studies, specifically of systematic reviews and randomized control trials, concerns were raised regarding the selection of participants, the limitations of the study designs, and the overall conclusion made regarding the results [5]. Additionally, it was recognized that while qualitative studies and their significance were addressed, the main studies brought into focus within the DCMS report were studies that followed the medical model standards of evidence (randomized control trials and systematic reviews) [5]. Discrimination of studies based on study design caused a dilution in quality and an omission of robust evidence derived from qualitative studies and narrative testimonies. Finally, in alignment with their past critics, Munira Mirza and Dr. Eleonora Belfiore, Clift and colleagues highlighted the lack of attention and scrutiny given to the interventions themselves, questioning how the quality of the therapies used, the role of the professional artists and the aesthetic criteria were assessed [5,6].

Many researchers shared the same opinions as Clift, Mirza and Belfiore, stating that results from music-based studies were divergent and inconclusive. However, integrative reviews have addressed the problem and pinpointed the root cause to be within the methodology of these studies [7,8]. One review, investigating music interventions in an oncology setting, flagged studies for experimenter bias, when researchers acted as therapists as well. This not only would influence the answers of the research participants themselves, but also the selection of data [7]. Another review of music interventions in the pediatric population recognized significant gaps in eight areas of intervention reporting, some of which include: intervention materials, intervention components, and intervention delivery schedules [8]. Insufficient information on the music interventions used prevents proper interpretation of the results: the first roadblock in the translation process of research to practice in a healthcare setting. Henceforth, to address the lack of critical appraisal of music-based intervention as a reliable research methodology, our research study utilized two quality assessment tools to investigate the quality of research within a subsection of studies within the WHO scoping review [2]. The studies used for this analysis were those that utilized music-based interventions (singing, listening, playing an instrument, active music making, Dalcroze Eurythmics) from the topic “Prevention of Ill Health”. 

## 2. Methods 

**Figure 1:**
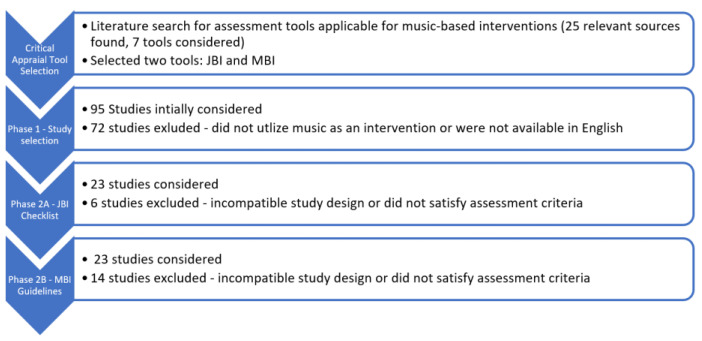
Flowchart of methodological process.

A quality assessment tool, also referred to as an appraisal tool or a study design evaluation tool, is a checklist or procedure that analyzes and assesses the methodological quality of a study design. To select two assessments tools that would best appraise all the different study designs within the WHO scoping review as well as the music-based interventions, a literature review was conducted. Once a manageable and concise list was curated, an analysis was done between the different tools that were compiled. A final list of 25 sources was created, and the assessment tools considered were: JBI checklist, MBI checklist, ROB assessment tool (Version 2), Jadad score, Newcastle-Ottawa Scale, Grade Recommendation Assessment Development of Evaluation (GRADE), PRISMA, AMSTAR 2 and CASP. After careful evaluation, it was decided that the assessment tool created by the Joanna Briggs Institute (JBI) was best equipped to appraise multiple studies within the WHO scoping review [9,10]. The JBI critical appraisal tools consisted of thirteen checklists, each checklist dedicated to assessing one of the following study designs: cross-sectional studies, case-control studies, case reports, case series, cohort studies, diagnostic test accuracy studies, economic evaluations, prevalence studies, qualitative research, quasi-experimental randomized control trials, systematic reviews, and text and opinion studies [9,10]. A second appraisal tool, the Music Based Intervention (MBI) Guidelines, was selected to assess the quality of the music interventions by assessing eleven items in relation to intervention reporting [11]. Please refer to Figure 1 for a visual representation and breakdown of the assessment process. 

Once these two critical appraisal tools were selected, the analysis was carried out in two phases. Phase 1 consisted of selecting the music-based studies within the preventative health category. Phase 2 focused on critically appraising the selected studies. Phase 2 was divided into Phase 2A and 2B since studies were assessed against both the JBI checklists and the MBI guidelines. The primary investigator oversaw training, data collection and final analysis. A second reviewer was recruited for Phase 2 to conduct a full-text analysis of the studies with both assessment tools. All raw data was inputted into a Google Excel sheet. All research assistants received the same training to ensure that the data collected was done in the same manner to ensure consistency and accuracy. 

Phase 1. The objective of Phase 1 was to organize all the music-based studies under “Prevention of Ill health” within the WHO report. The data gathered from individual researchers were shared on a Google Excel sheet, to which the primary investigator had access. Tutorials and additional documents were prepared by the primary investigator to help explain the protocol for selection and to provide the research team with information on how to organize the studies based on music intervention and study design. The information obtained from the study included the citation, the form of art therapy used (visual art, music, dance, etc.), the type of study design, and the genre of music (if applicable). A colored legend was created and remained consistent throughout the data collection process. The legend was used to help identify studies that required further investigation, that were unable to be found, or for which no translation was available. The studies had to fall under one of the following categories: case-control, cross-sectional, longitudinal cohort, qualitative, quasi-experimental, randomized control trials, and systematic/meta-analysis. If a study did not match the study designs listed, it was marked as ‘other’. Once all the studies were categorized, the finalized list was curated by filtering for studies that utilized music-based interventions. 

Phase 2A. Based on the finalized list from Phase 1, studies were evaluated in correspondence to the JBI checklist. The primary investigator analyzed each study with the appropriate checklist. The information from these analyses was collected on a Google Excel sheet. A legend with the commonly used terms and their abbreviations was created to ensure that the assessment formats remained consistent. Each item was answered by four options, Yes (Y), No (N), Unclear (U), or Not Applicable (N/A). The same evaluation was completed by a second reviewer, on a separate Excel sheet, who assessed 15% of the studies listed. 

Phase 2B. Phase 2B utilized the Music Based Intervention Guidelines. The data was recorded on a separate Google Excel sheet. Unlike Phase 2A, where certain studies were omitted for their study design, Phase 2B accommodated all study designs so long as an appropriate music intervention was utilized. This second appraisal focused on the variables that affected the quality of the music intervention delivered, rather than on the components of the study design. To remain consistent, the same legend utilized in Phase 2A was utilized here as well: each item was answered by four options, Yes (Y), No (N), Unclear (U), or Not Applicable (N/A). The same evaluation was completed by a second reviewer, on a separate Excel sheet, who assessed 15% of the studies listed. 

Appraisal Scores. All appraisal scores from Phase 2A and Phase 2B were digitally input into an Excel sheet. Appraisal scores were not used to grade or rank the studies, but rather to provide a numerical value to understand the general strength of the study design and the intervention used. This was done to ensure that those studies not required to meet certain criteria were not held at a disadvantage during the final analysis. 

## 3. Results

Phase 1. Under “Prevention and Promotion”, the topic of interest was “Prevention of Ill health”, which focused on six health issues: Wellbeing, Mental Health, Trauma, Cognitive Decline, Frailty and Premature Mortality. A total of 95 studies were included, and 23 studies that utilized music as an intervention and were available in English were selected for Phase 2. There were zero music studies within the subtopic “Trauma”, while the subtopic “Cognitive Decline” had the most music-related studies. See Table 1 for a breakdown of the number of studies within each of the six health issues.

Phase 2. Of the 23 studies selected for analysis, four studies were not eligible for both the JBI and MBI assessment, because the study designs (for example, bibliographic review) were not compatible with the critical appraisal checklists available. Of the remaining 19 studies, seven received a full analysis, given that a music intervention was utilized and the study design was accepted within the JBI critical appraisal tool checklists. The remaining 12 studies received partial assessment based on which assessment tool criteria was met. Studies that did not satisfy the criteria required for assessment were marked with N/A (not applicable) to denote incomplete assessment. Table 2 shows a breakdown of the types of studies within each of the six health issues, and Table 3 shows the final JBI and MBI scores for the studies that underwent the evaluation. 

## 4. Discussion 

Through this critical appraisal, most of the studies that were assessed by the JBI checklists received moderate scores, indicating that most of the criteria were met regardless of study design. However, when compared against the MBI guidelines, the majority of the studies received low scores, highlighting the fact that most of the music-based interventions within the studies failed to report important variables essential to the overall validity of the study results and the quality of the research design. In contrast, the DCMS Policy Review stated that research within the topics of “Wellbeing”, “Prevention of physical illness in adults” and “Cognitive decline” received an evaluation of Grades A and B [3]. This grading signifies that the research body within these topics is of high value and could be utilized in policy guidelines and practices [3,4]. However, the findings from our critical review support the concerns originally addressed by Clift and colleagues [5] in their critique regarding the DCMS Policy Review. Another aspect of the DCMS policy review that is challenged by the findings of our research is the significance and quality of randomized control trials (RCT). Of the three randomized control trials included in our assessment, only one study [31] met most of the criteria for both the JBI and MBI checklists. The remaining two randomized control trials [26,28] fared poorly against the MBI guidelines, fulfilling less than 30% of the criteria. The methodologies in both research studies provided insufficient details on the type of music used, the intervention protocol and the strategies. Hence, our findings refute the claims made by the DCMS policy review regarding the significance of randomized control trials over qualitative research and other study designs [3]. 

Despite the low scores utilizing the MBI guidelines, it is important to note that these studies lacked the ability to blind the participants of the music intervention studies, an item that was assessed in the JBI checklist. In randomized control trials, blinding refers to the process of masking the original and true contents of the treatment from either the participant, the researcher, or both [35]. The method of blinding the participants reduces bias by prohibiting a phenomenon known as the “placebo effect”. The placebo effect is when participants who receive the fake/inactive medication believe that their health or symptoms are improving [35]. This group usually acts as the control group. However, this process was not possible in music-based randomized control trials, since the treatment offered was an intervention rather than a medication. Thus, participants could be randomized to the group (treatment or control), but they became aware of their category once they started the intervention. This resulted in many studies having the item on the RCT checklist evaluated as “Not applicable.” In this way, the study is not penalized for omitting this criterion. However, in randomized control trials, aside from randomization, the process of blinding holds a lot of value in reducing the risk of bias. Thus, once a participant is aware of their treatment they may alter how they respond to the treatment, leading to skewed results. In response to this, the study should ensure that the interventionist and the researcher are blinded. This was the case in many studies, showing some integrity to the RCT study design features.

While our findings did not support the DCMS policy report, our research did provide a detailed analysis to highlight commonly missed criteria and the effects of such on the results presented. For instance, a common trend seen in all the studies that were assessed was the lack of treatment fidelity. The MBI checklist states that treatment fidelity “describes strategies used to ensure that treatment and/or control conditions were delivered as intended” [11]. This includes intervention protocols and monitoring. The importance of this criteria is based on the idea of consistency within the study design protocol to ensure that, even with different research assistants or interventionists, the methodology by which the study was administrated would not produce skewed results [11]. Studies failed to mention any training protocols taken by the interventionist when conducting the intervention, especially when there was a lack of information on the interventionist’s qualifications.

Interventionist qualifications were assessed under a separate item, to ensure that the individual selected was knowledgeable and qualified to conduct the therapy. This criterion was especially important in music interventions based on playing, learning or listening to music. For example, the randomized control trial by Trombetti and colleagues [28] investigated Dalcroze Eurhythmics, a concept commonly used in music education to help educate students about rhythm through movement [36]. Given that this intervention takes a unique approach to movement that differs from dance concepts such as contemporary and ballroom, an instructor teaching Dalcroze Eurhythmics should have the proper certifications and training. However, within the study methodology, all that was mentioned was that an “experienced instructor” conducted the lesson. The lack of credentials and details such as educational background bring into question whether the instruction given was effective and conceptually correct. 

A similar concern was recognized in a study that analyzed the influence of music listening on risk of falls in the geriatric population, where participants were to listen to a performance held by volunteer musicians [32]. The only information provided about these musicians, who would be playing an hour session for the purpose of this study, was that they were students from McGill who played piano, guitar, saxophone, and violin, or were singers [32]. No further indication was made regarding their music proficiency, or if all these students had completed the same level of training. This would have been an important factor to consider, since the volunteer musicians’ proficiency level would affect their repertoire selection, their ability to engage with the audience, and their overall experience as they deliver the hour session. Each of these components are variables that could modify the results if not accounted for, as it could influence the number of sessions attended by the patient. It was mentioned in the study that the musicians put on four one-hour sessions per week. If a certain group had better engagement than another, or played a more favorable selection of music, then participants in the study would be more inclined to only attend those sessions, since attendance was voluntary. This was another example of how important it is to not only state these variables within the research, but to also address them in the research study as possible confounding variables.

In comparison to the studies mentioned above, there were two studies that received high appraisal scores through the MBI checklists as well as through the JBI checklists. The first study, as previously mentioned, was a randomized control trial utilizing rhythmic auditory stimulation which allowed the synchronization of gait movement to music embedded with a metronome click [31]. Aside from the criteria necessary for a strong randomized control trial, the study also included the genre of music (folk/classical), the delivery method (live or headphones), the tempo of the music (2/4), and stated the protocol and training that participants underwent before beginning the intervention [31]. The second study to receive a high appraisal score was a qualitive study utilizing a drumming intervention [12]. The drumming intervention was administered as a multi-dimensional approach to enhance the wellbeing of caregivers [12]. The study conducted interpretive phenomenological analysis on the findings from interviews conducted after the intervention was administrated [12]. The intervention was conducted in weekly sessions by a professional drummer from the Royal College of Music [12]. Details pertaining to the structure of the class, the seating arrangement, the content being taught, and the frequency of the lessons were provided in detail. All factors that could act as confounding variables were accounted for, thus the results reported could be considered of high quality. Therefore, by utilizing these studies as exemplars, future research can ensure that similar errors and risk of biases can be overcome, creating the potential to support the implantation of policy guidelines. 

### 4.1. Challenges and Limitations 

A common challenge experienced by the primary investigator during Phase 1 involved the classification of a study’s design/methodology. When the study design used was not explicitly stated, researchers utilized information provided within the abstract and methodology to help identify the study design. Another limitation to address within Phase 2 was the lack of assessment tools available for certain study designs. As mentioned previously, the JBI critical appraisal tools were selected due to their ability to cater to a wide range of qualitative and quantitative study designs. However, during our analysis, we flagged several review papers that were incompatible with both the MBI and JBI checklists. Studies were incompatible if the study design did not fall under one of the categories within the JBI checklists, or if a music intervention was not utilized. An example of such a study design would be reviews, of which there are 14 different types [37]. Two of the review types accounted for in the JBI checklists are systematic reviews and meta-analyses, which can be assessed using the same checklist. However, for reviews such as literature reviews, scoping reviews, overviews, and critical reviews, there were no checklists within the JBI critical appraisal tools accounting for these designs. Hence, these studies were excluded from the final critical appraisal because of the lack of assessment tools available. This limitation creates a discrepancy within the data gathered, as many of the studies investigated within these reviews were also omitted. For example, a literature review investigating the effect of sound interventions on physical and mental healthcare in the clinical environment analyzed 266 studies [38]. The information offered by these studies should be investigated on an individual basis to properly account for internal validity and data reliability. Additionally, an inherent limitation faced within our study design was the inability to include research studies conducted from 2020 and on. Given that our study focused on studies within the WHO scoping review, future research investigating the quality and effectiveness of music interventions should include present research. 

### 4.2. Recommendations and Next Steps 

While our research investigates the components of music intervention, very little attention is given to the mechanism of effect of music interventions. The studies reviewed in this evaluation were primarily evidence-based, thus determining whether or not music interventions were effective, yet very rarely investigating why they were effective. More recently, researchers are starting to explore this question. In a paper by Clements-Cortes and Bartel, a model of mechanism response was created to help understand how various music therapies work. The model investigated the effects of learned cognitive response, learned isomorphic and primal response, cognitive activation of neural circuits, stimulated neural coherence, and effects on the cellular/genetic level [39]. For example, in a study investigating the long-term effects of 40 Hz physio-acoustic vibrations on motor impairment in Parkinson’s disease, the mechanism of response was investigated at the neural level. Understanding the mechanism of deep brain stimulation allowed this study to replace invasive treatment with vibrations, since this was determined to stimulate similar effects, where pathological oscillatory activity associated with PD was disrupted [40]. Additionally, according to Bartel and Mosabbir, a focus on mechanism not only allows therapists to make use of enhanced knowledge and awareness of mechanism to maximize clinical effect, but also aids in legitimizing the approach in the scientific community, leading to expanded use in healthcare [41]. Therefore, assessing studies for the mechanism of response and encouraging future research to explore this aspect could be highly beneficial in providing quality studies for evidence-based healthcare. 

Another recommendation for future research would be the inclusion of information on the population being tested. This data should be considered when assessing the validity and reliability of a study’s results. Sample size and gender are two factors that significantly impact the quality, eligibility, and relatability of the results. Sample size in many studies is used to represent a larger population of interest given that research on every individual (such as a consensus) is not practical or possible [42]. Hence, the sample population used in studies and the size of the study population are important, as the findings from the research conducted on this group of individuals may be generalized for a large population. Additionally, it is worthwhile to acknowledge the differences in representation in terms of gender. Not only has there been an increased demand for research on women’s health, but research has shown that gender is a factor that can affect results from a biological, physiological, and mental standpoint [43]. Hence, by accounting for both sample size and gender, we can address the gaps in the scientific literature from a different angle.

Another avenue to explore would be music in various cultures. The evaluations using the MBI tools brought to light a lack of diversity in the music genres in the studies analyzed. Broadening the scope from Western music would allow researchers to investigate music traditions, elements, and practices within different cultures. Certain cultures have a rich history of music in health, where music has been used as a healing technique for many years. For example, indigenous communities around the world have long traditions of the arts being embedded into their everyday life [44]. The arts, in various forms, have been used holistically in healing [44]. Today, many community-based programs have been established to help aid in the healing journeys of many indigenous communities [45]. However, scoping reviews such as the WHO report miss much of the research that is available on such topics, given that only one research article was included regarding indigenous communities and music. It would be beneficial to consider topics such as indigenous health, and to conduct in-depth reviews to understand what literature is available and what further research can be done.

## 5. Conclusions 

The WHO scoping review by Fancourt and Finn [2] is only the beginning of understanding the depth of research incorporating health and music. Given the broad scope of the review, the number of diseases and health issues addressed, and the various interventions included, there is a need for a more rigorous, specific, and systematic review to be conducted. In order for proper policies to be implemented using music therapies and interventions on a public health level, it is vital that the evidence-based research guidelines are derived from research that comes from studies with valid and reliable results, study designs and methods. Our paper sheds light on the importance of assessing studies for their quality and effective methodology, which inadvertently alters the reliability and the validity of the outcomes presented. The use of the JBI and MBI appraisal tools has allowed the research team to explore and curate an understanding of which criteria were commonly overlooked and which studies could act as paradigms for future research. The review demonstrates that several studies did not fulfill criteria that directly impacted the replicability and reproducibility of the research. Thus, it is evident that a novel study that addresses the identified gaps in literature and reproduces reliable data will provide new insights, and will ensure that the relationship between music and health is indisputable. While the goals outlined by the DCMS policy review [3] are unattainable now, utilizing music-based assessment tools, such as the MBI guidelines, to conduct reviews in line with the opinions stated in the review by Clift and colleagues [5], would yield great benefits for music and health research. 

## Figures and Tables

**Table 1:** Summary of results from Phase 1 Topic: Prevention and Promotion.

Topic (Prevention and Promotion)	Total Number of Studies	Number of Music Studies	Number of Missing Music Studies *	Number of Music Studies Excluded (No CA) **
Prevention of Ill Health	95	24	1	9
Wellbeing	21	4	0	2
Mental Health	16	4	0	1
Cognitive Decline	23	9	1	3
Frailty	24	5	0	1
Trauma	0	0	0	0
Premature Mortality	11	2	0	2

* Missing studies were counted if the type of intervention was music; missing studies with art/dance/other interventions were not included. ** Excluded studies refers to study designs that did not match the criteria for critical appraisal by the JBI Checklists. These studies were not part of Phase 2A analysis.

**Table 2:** Summary of results from Phase 2A Topic: Prevention and Promotion.

Topic (Prevention)	Number of Music Studies	Case-Control	Case Study	Case Series	Cohort	Cross-Sectional	Qualitative	Quasi-Exp.	RCT	Sys Rev/MA	Other
Prevention of Ill Health	20										
Wellbeing	4				1		1			1	1
Mental Health	4						1			1	2
Cognitive Decline	9	3			2				1	1	2
Frailty	5				1			1	2	1	
Trauma	0										
Premature Mortality	2										2

Other: The studies were excluded from the analysis due to the lack of compatibility between the JBI checklists and the study design.

**Table 3:** Summary of results from Phase 2 A&B Topic: Prevention of Ill Health.

Prevention of Ill Health	Study Design	Music Intervention	JBI Score	MBI Score
Wellbeing				
Ascenso et al., 2018 [12]	Qualitative Research	Drumming Intervention	8/10	9/11
Daykin et al., 2017 [13]	Systematic Review	Singing	10/11	N/A
Hallam et al., 2016 [14]	Cohort Study	Music Making	1/11	1/11
Papinczak et al., 2015 [15]	Mixed Method (qualitative and cross sectional)	Q: N/A C: N/A	Q: 7/10 C: 3/8	Q: N/A C: N/A
Mental Health				
Linnemann et al., 2018 [16]	Exploratory Ambulatory Assessment	Music Listening	N/A	2/11
Linnemann et al., 2016 [17]	Exploratory Ambulatory Assessment	Music Listening	N/A	2/11
Panteleeva et al., 2017 [18]	Meta Analysis	Music Listening	9/11	N/A
Cognitive Decline				
Strong and Mast (2018) [19]	Cohort	N/A	7/9	N/A
Gooding et al., (2014) [20]	Cohort	N/A	7/11	N/A
Schneider et al., (2018) [21]	Scoping Review	N/A	N/A	N/A
Moussard et al., (2016) [22]	Case-Control Study	N/A	7/10	N/A
Dawson (2014) [23]	Bibliographic Review	N/A	N/A	N/A
Balbag et al., (2014) [24]	Case-Control Study	N/A	10/10	N/A
Kim and Yoo (2019) [25]	Systematic Review	Instrument Playing	10/11	N/A
Degé and Kerkovius (2018) [26]	Randomized Control Trial	Drumming	9/12	2/11
Moreno-Gomez et al., (2017) [27]	Case-Control Study	N/A	4/10	N/A
Frailty				
Trombetti et al., 2011 [28]	Randomized Control Trial	Music Training	10/12	3/11
Ghai, Ghai, and Effenberg (2018) [29]	Systematic Review	Rhythmic Auditory Cueing	10/11	N/A
Coste et al., 2018 [30]	Quasi-experimental	Stand/Sway To Beat	5/9	4/9
Thaut et al., 2018 [31]	Randomized Control Trial	Rhythmic Auditory Stimulation	9/12	10/11
Chabot et al., 2019 [32]	Cohort	Music Listening	6/10	3/11
Premature Mortality				
Dunbar (2003) [33]	Narrative Review	N/A	N/A	N/A
Mithen (2006) [34]	Book	N/A	N/A	N/A

N/A = not applicable; the studies in which both the JBI and MBI guidelines could not be used are still included, in order to keep track of the all the studies within this topic.

## Data Availability

Raw data from the critical assessment carried out in the methodology can be obtained through requests to the corresponding author.
